# Malnutrition among Cancer Patients in a Tertiary Care Centre: A Descriptive Cross-sectional Study

**DOI:** 10.31729/jnma.7616

**Published:** 2022-11-30

**Authors:** Anuj K.C., Bishnu Dutta Poudel, Ramila Shilpakar, Sudip Thapa, Rajiv Sharma, Bibek Acharya, Sandhya Chapagain, Ambuj Karn, Saugat Poudel, Bishal Paudel

**Affiliations:** 1 Department of Medical Oncology, Bhaktapur Cancer Hospital, Dudhapati, Bhaktapur, Nepal; 2 Department of Clinical Oncology, National Academy of Medical Sciences, Mahaboudha, Kathmandu, Nepal; 3 Department of Haematology, Civil Service Hospital, New Baneshwor, Kathmandu, Nepal

**Keywords:** *malnourishment*, *nutritional deficiency*, *screening*

## Abstract

**Introduction:**

Malnutrition is one of the most frequent disorders among cancer patients. It is seen in 50-90% of cancer patients. This high prevalence of malnutrition is very concerning as it is associated with reduced effective treatment, functional status, quality of life and survival. The aim of the study was to find out the prevalence of malnutrition among cancer patients in a tertiary care centre.

**Methods:**

A descriptive cross-sectional study was conducted among 95 cancer patients in the Department of Clinical Oncology of a tertiary care centre from 25 January 2022 to 25 July 2022. Ethical approval was obtained from the Institutional Review Committee (Reference number: 1192/2078/79). Convenience sampling was done. Patients were screened using Patient-Generated Subjective Global Assessment for malnutrition. Point estimate and 95% Confidence Interval were calculated.

**Results:**

Among 95 cancer patients, 22 (23.15%) (15.10-32.90, 95% Confidence Interval) were malnourished.

**Conclusions:**

The prevalence of malnutrition was found to be lower than in other studies done in similar settings. Nutritional assessment and support should be an integral part of care for gastrointestinal cancer.

## INTRODUCTION

Malnutrition is common in cancer patients accounting for 50-90%.^1^ It is associated with reduced effective treatment, functional status, quality of life, and survival.^2^ Its prevalence varies with type, site and stage of cancer with the highest being in cancer of the gastrointestinal tract and lung.^3^ It also occurs as a result of various factors including reduced food intake, and adverse effects of chemotherapy.^4^

Patients with malnutrition have reduced tolerance to antineoplastic therapy^2^ with more dose-limiting toxicity and treatment interruptions, thus requiring more frequent and longer hospital admissions.^5^

The objective of the study was to find out the prevalence of malnutrition among cancer patients in a tertiary care centre.

## METHODS

A descriptive cross-sectional study was conducted among 95 cancer patients in the Department of Clinical Oncology of the National Academy of Medical Sciences (NAMS) from 25 January 2022 to 25 July 2022. The ethical approval was obtained from the Institutional Review Committee of NAMS (Reference number: 1192/2078/79). Each patient was explained about the study and consent was taken.

All the patients of any age, diagnosed with cancer of any organ, presenting to the clinical oncology department of NAMS, were included. Patients who were already on nutritional support and with difficulty in communication such as dementia or altered sensorium were excluded. The sample size was calculated by using the formula:


$$\begin{array}{ll} \text{n} & ={\text{Z}}^{2}\times \frac{\text{p}\times \text{q}}{{\text{e}}^{\text{2}}}\\ & =1.96^{2}\times \frac{0.435\times 0.565}{0.10^{2}}\\ & =95 \end{array}$$

Where,

n= minimum required sample size
Z= 1.96 at 95% Confidence Interval (CI)
p= prevalence of malnutrition from the previous study, 43.5%^6^
q= 1-p
e= margin of error, 10%

As this worksheet contained two parts, each containing four questionnaires, the first part was filled up by eligible patients themselves. As this form was unavailable in the Nepali version, patients who couldn't read English were assisted for completion. After completing all the data, the sum of the points of each component generating a score, ranging from 0 to 9 was calculated. According to the numerical score, PG-SGA presents different types of nutritional interventions, where: 0 to 1 point no nutritional intervention was required; between 2 and 3 points, nutritional education of the patient or family was suggested; between 4 and 8 points nutritional intervention by a nutritionist was required, and >9 points indicated a critical need for symptom control and nutritional interventions. Those patients who required nutritional support were given according to the standard of care. In addition to quantitative classification, a classification by groups was performed, in which the patients were classified as: well-nourished or anabolic (SGA-A); moderately malnourished or suspected of malnutrition (SGA-B); or severely malnourished (SGA-C).

Data were entered and analysed in IBM SPSS Statistics 20.0. Point estimate and 95% Confidence Interval were calculated.

## RESULTS

Among 95 patients, 22 (23.15%) (15.10-32.10, 95% CI) of the patients were malnourished. Among them, 14 (14.90%) patients were moderately malnourished or had suspected malnutrition SGA-B and 8 (8.50%) patients were severely malnourished SGA-C. A median PG-SGA score of 2 (Interquartile range:1-6) was present.

When classified based on the type of nutrition intervention requirement, a total of 22 (23.15%) patients required some intervention for their malnutrition. The study also indicated that 16 (72.72%) of the patients had a score of >9 which implies a critical need for improved symptom management and/or nutrition intervention. Out of 16 patients, 8 (50%) were in SGA-B and 8 (50%) in the SGA-C group. Six (27.28%) patients had a score of less than 9, which implies a requirement of intervention by a nutritionist in conjunction with a nurse or physician.

Among 22 patients, 18 (81.81)%) patients who were diagnosed as having some sort of gastrointestinal cancer had malnutrition. Out of total malnutrition patients, 14 (63.63%) of them had the metastatic form of cancer (Table 1).

**Table 1:** Demographic profile malnutrition (n= 22).

Variables	n (%)
**Sex**	
Male	18 (81.81)
Female	4 (18.19)
**Diagnosis**	
Gastrointestinal cancers	18 (81.81)
Lungs	2 (9.09)
Non hodgkins lymphoma	1 (4.54)
Ovary	1 (4.54)
**Stage**	
Limited	8 (36.36)
Metastatic	14 (63.63)
**Malnutrition grading**	
PGSGA-B	14 (63.63)
PGSGA-C	8 (36.36)

## DISCUSSION

This study assessed malnutrition in 95 patients visiting the tertiary cancer centre of Nepal using the PG-SGA score. The prevalence of malnutrition in our study was 23.15%, whereas in Kenyan cancer patients it was 31%.^7^ Moreover, among the total malnutrition patients in our study, 14.90% were moderately malnourished (SGA-B) and 8.50% severely malnourished (SGA-C) whereas 19.70% and 11.30% were moderately and severely malnourished in Kenyan patients. This highlights the difference in nutrition status between Asian and African populations with cancer. These differences in results might be due to different methodologies, culture and also most of the Kenyan patients were of advanced stage.

Our study showed that 50% of patients with gastrointestinal cancer including head and neck cancer had moderate or severe malnutrition. A study done in Mexico showed the highest prevalence of malnutrition among patients with lungs followed by head and neck and gastrointestinal cancers.^8^ The reason might be gastrointestinal cancers which affect absorption, lead to dysphagia, vomiting, diarrhoea and eventually lead to malnutrition.

Malnutrition was higher in metastatic cancer.^7^ In our result 33.33% of patients had malnutrition in comparison to the Kenyan study (24.30%). Metastatic cancer is chronic and incurable stage of disease, where patients might have received multiple lines of chemotherapies and also due to chronic illness which might have contributed to malnutrition. As this scoring relies mostly on the dietary habits for two weeks, we need serial measurements during the course of illness as this screening tool stratifies those who are at risk and can be managed accordingly.

## CONCLUSIONS

The prevalence of malnutrition among cancer patients was found to be lower than in other studies done in similar settings. Malnutrition is a common problem seen in cancer patients. Therefore, early recognition and detection of risk for malnutrition, especially gastrointestinal cancer, through screening followed by comprehensive assessment and timely interventions should be an oncology strategy. Furthermore, nutritional support should be an integral part of cancer care.
